# Immunoinformatics Design and Assessment of a Multiepitope Antigen (OvMCBL02) for Onchocerciasis Diagnosis and Monitoring

**DOI:** 10.3390/diagnostics12061440

**Published:** 2022-06-11

**Authors:** Bernis Neneyoh Yengo, Cabirou Mounchili Shintouo, An Hotterbeekx, Ntang Emmaculate Yaah, Robert Adamu Shey, Jusal Quanico, Geert Baggerman, Lawrence Ayong, Luc Vanhamme, Rose Njemini, Jacob Souopgui, Robert Colebunders, Stephen Mbigha Ghogomu

**Affiliations:** 1 Department of Biochemistry and Molecular Biology, Faculty of Science, University of Buea, Buea P.O. Box 63, Cameroon; bernisyengo@gmail.com (B.N.Y.); cabirou.mounchili.shintouo@vub.be (C.M.S.); mayaahemma@gmail.com (N.E.Y.); sheynce@gmail.com (R.A.S.); 2 Department of Gerontology, Faculty of Medicine and Pharmacy, Vrije Universiteit Brussel, Laarbeeklaan 103, 1090 Brussels, Belgium; rose.njemini@vub.be; 3 Frailty in Ageing Research Group, Vrije Universiteit Brussel, Laarbeeklaan 103, 1090 Brussels, Belgium; 4 Global Health Institute, University of Antwerp, 2610 Antwerp, Belgium; an.hotterbeekx@uantwerpen.be (A.H.); robert.colebunders@uantwerpen.be (R.C.); 5 Molecular Pathology Group, Laboratory of Cell biology and Histology, University of Antwerp, 2610 Antwerp, Belgium; 6 Center for Proteomics, University of Antwerp, Groenenborgerlaan 171, 2020 Antwerpen, Belgium; jusal.quanico@uantwerpen.be (J.Q.); geert.baggerman@uantwerpen.be (G.B.); 7 Malaria Research Unit, Centre Pasteur Cameroon, Yaoundé P.O. Box 1274, Cameroon; ayong@pasteur-yaounde.org; 8 Department of Molecular Biology, Institute of Biology and Molecular Medicine, IBMM, Gosselies Campus, Université Libre de Bruxelles, 1070 Brussels, Belgium; luc.vanhamme@ulb.be (L.V.); jsouopgu@ulb.ac.be (J.S.)

**Keywords:** onchocerciasis, OvMCBL02, multiepitope antigen, IgG, diagnosis

## Abstract

Onchocerciasis is a Neglected Tropical Disease that has a significant socioeconomic impact, especially in Sub-Saharan Africa. Numerous reports indicate that the Expanded Special Project for the Elimination of Neglected Tropical Diseases needs novel diagnostic tools before achieving its goal of successful elimination of onchocerciasis in Africa. The current diagnostic tests are either invasive, insensitive, or not applicable in the field and about 25% of persons infected cannot mount immune responses against the single antigen used in the only approved Ov-16 serological test. In the quest to identify novel biomarkers that can be used to certify that a patient is free from the disease, evaluate the progress of elimination programmes, and conduct post elimination surveillances, mass spectrometric analysis of *Onchocerca volvulus* crude extract revealed that 1392 proteins are expressed in the adult and microfilariae stages of the parasite. Computational analysis predicted six of the proteins as *O. volvulus* potential diagnostic targets. Linear B-epitopes were predicted from the six proteins and used to construct a multiepitope antigen (OvMCBL02). Serological analysis revealed that the OvMCBL02 test significantly differentiated between serum samples of onchocerciasis patients from the Kombone Health Area in the South West Region of Cameroon (*n* = 63) and control serum samples from Rwanda (*n* = 29) and Europe (*n* = 26) as well as between serum samples from the onchocerciasis hyperendemic region of Kombone Health Area (*n* = 63) and the hypoendemic region of Bandjoun Health District (*n* = 54). Interestingly, the test did not cross-react with serum samples from patients suffering from related nematode infections, thereby suggesting that further characterization of the OvMCBL02 multiepitope antigen will render it an additional member of the diagnostic toolbox for the elimination of onchocerciasis.

## 1. Introduction

Human onchocerciasis, also known as “river blindness”, is caused by the parasitic worm *Onchocerca volvulus* (*O. volvulus*). The parasite is transmitted to humans through exposure to repeated bites of female blackflies that belong to the genus *Simulium* [1]. The disease, which manifests primarily as eye and skin lesions [2,3], has a significant socioeconomic and public health impact [4]. The patients are susceptible to HIV infection [5], epilepsy [6], and the disease is also a potential risk for glaucoma [7] in remote regions of Africa and Latin America. The latest Global Burden of Disease Study conducted in 2017 revealed that a minimum of 220 million individuals needed preventive chemotherapy against onchocerciasis. Furthermore, 69.8% and 5.5% of the 20.9 million infected individuals (more than 99% living in Africa) already have skin disease and vision loss respectively [1].

To alleviate the burden of onchocerciasis, the Expanded Special Project for Elimination of Neglected Tropical Diseases (ESPEN) was created by the WHO to eliminate the disease in Africa. ESPEN relies on population-based treatment with ivermectin to eliminate onchocerciasis, with a minimum requirement of 80% therapeutic coverage which has to go on for about 15 years of yearly treatment corresponding to the lifespan of adult *O. volvulus* worms [8]. For ESPEN to differentiate its activities from those of former onchocerciasis control programmes, it must include all areas where *O. volvulus* is currently being transmitted in its elimination plans. Thus, the diagnosis of onchocerciasis patients is the first step to be performed by ESPEN. The second step, which lasts for about 15 years, corresponds to the treatment cycle of ESPEN where monitoring of the treatment plan is essential as well as determining when to certify an individual to be free from the disease. The last step is the post-treatment surveillance that needs to be conducted to prevent recurrence of the disease. Thus, the objectives of ESPEN largely depend on a highly sensitive and specific diagnostic test for detection of the parasite in humans.

Detecting microfilariae in skin snips has been the method of choice for human onchocerciasis diagnosis. This method is known to be highly specific, but unfortunately it is invasive, inflictive, and insensitive when a patient’s microfilaria load is low. Although PCR-amplification of *O. volvulus* DNA can increase its sensitivity, and the method is ineffective to diagnose prepatent infection [9]. Efforts have also been undertaken to create novel diagnostic tests based on *O. volvulus* metabolites [10,11] and circulating nucleic acid in skin biopsies [12,13]. Nevertheless, these techniques are not sensitive enough to be recommended for use [14,15]. Additionally, other investigations combining high throughput genomic with transcriptome and proteomic approaches have been carried out [16,17]. At the moment, the WHO has authorized only the Ov-16 ELISA test to verify interruption of transmission of the parasite, monitor elimination programs, and possible recrudescence. Nonetheless, the test is limited by its inability to diagnose about 20% of the onchocerciasis infection [18]. Hence, there is the need for continuous search for more robust diagnostic tools for detection and sero-surveillance of onchocerciasis. Multiepitope chimeric antigens have been reported to have higher diagnostic values in diagnosis with potentials characteristic of higher sensitivity and specificity [19] such as in the serodiagnosis of nematode infections [20,21,22], hepatitis C virus [23], toxoplasmosis [24,25], HIV-1 [26,27], and Chagas disease [28]. Hence, in this work, we set out to design a novel diagnostic biomarker (OvMCBL02 multiepitope antigen) using proteomics and immunoinformatics tools and assess its potential for the diagnosis of onchocerciasis using indirect ELISA.

## 2. Materials and Methods

### 2.1. Ethical Consideration

This work was approved by the Cameroon Bioethics Initiative (CAMBIN) Ethics Review and Consultancy Committee (ERCC) (approval number: CBI/443/ERCC/CAMBIN). All persons who voluntarily agreed to be part of this study were given informed consent forms which they signed after explicit explanations of what the work would entail. It was ensured during data collection, processing, and reporting that participants’ privacy was protected.

### 2.2. Study Site, Population and Sample Collection

A trained and certified medical practitioner examined all participants before collecting blood and nodule samples from patients residing in the onchocerciasis endemic region of Kombone Health Area in the South West Region of Cameroon. These *O. volvulus* infected individuals (OVS, *n* = 63) were selected based on the presence of skin and eye lesions associated with onchocerciasis or microfilaria in skin biopsies and/or nodules. A maximum of 2 nodules were excised from each patient into sterile 25 mL RPMI 1640 medium under aseptic conditions and transported to the Molecular and Cell Biology Laboratory, University of Buea, Cameroon. Adult worms were obtained from nodules as described by Schulz-Key et al. [29]. Briefly, each nodule was cultured at 37 °C in 10 mL RPMI-1640 medium (Thermo Fisher, Merelbeke, Belgium) supplemented with 0.25 mg/mL of gentamicin sulphate (Sigma, St. Louis, MI, USA) and 0.5 mg/mL of collagenase (type 1) (Sigma, St. Louis, MI, USA) at 100 RPMI overnight to isolate the adult worms from the digested nodules. The adult worms were cultured for 24 h in 5% CO_2_ at 37 °C in 1 mL incomplete culture medium (RPMI-1640, supplemented with 25 mM HEPES, 2 g/L sodium bicarbonate, 2 mM L-glutamine, 150 μg/mL penicillin/streptomycin and 0.5 μg/mL amphotericin B pH 7.4) using 12-well plates to obtain microfilaria and excretory products. The adult worms, microfilariae, and excretory products were frozen and transported to the Global Health Institute, University of Antwerp, Belgium for proteomics analysis.

Serum samples were prepared from blood obtained from individuals that were microfilaria negative and did not have any clinical manifestation of *O. volvulus* infection in the hypoendemic region of Bandjoun Health District, West Region of Cameroon. Ivermectin has been administered to these individuals (ITS, *n* = 54) for more than 20 years and parasitological reports indicate that this Region is making significant progress to eliminate the disease [30]. Control serum samples were obtained from healthy persons living in Huye, Rwanda (HES, *n* = 29) reported as a low-risk zone for *O. volvulus* infection [31] and from Europeans (ECS, *n* = 26) who have never visited an onchocerciasis-endemic region. Serum samples from persons suffering from related nematode infections like *Wuchereria bancrofti* (WBS, *n* = 6), *Mansonella perstans* (MPS, *n* = 6), *Brugia malayi* (BMS, *n* = 3), or *Ascaris lumbricoides* (ALS, *n* = 6) were obtained from the Molecular Resources Division of the Filariasis Research Reagent Resource Center (Northampton, MA, USA).

### 2.3. Proteomic Profile of O. volvulus

The proteomic profiles of *O. volvulus* adult male and female, microfilaria, and their excretory products (collected after 3, 6 and 24 h of incubation) were generated by mass spectrometry using conventional shotgun proteomics [32]. Briefly, the worm samples were placed in lysis buffer containing 40 mM Tris, 6 M Urea and 1.5 M Thiourea, 66 mM tris-(2-carboxyethyl) phosphine (TCEP) and protease inhibitor cocktail. The adult samples were homogenized in lysis buffer (40 mM Tris, 6 M Urea, 1.5 M Thiourea) for 4 × 30 s and the microfilaria sample for 2 × 30 s. Then, 20 µg was taken from each sample, while 200 µL were taken from the homogenized worm samples for subsequent analysis. Following TCEP-mediated reduction, the cysteine residues were alkylated with 2-Iodoacetamide to a final concentration of 19 mM for 30 min in the dark. After incubation, the proteins were precipitated with ice-cold acetone overnight at −20 °C. Acetone was removed after centrifugation (8000× *g*, 10 min, 4 °C), and the pellet was dissolved in 100 mM Triethylammonium bicarbonate (TEAB) and deposited on filter-aided sample preparation (FASP) columns, and overnight digestion was performed by adding 4 µg of trypsin. The peptides were then desalted using C18 spin columns.

Each sample (0.5 μg) was loaded on a micropillar array (µPAC™) trapping column and injected on a 200 cm C18 µPAC™ column (Pharmafluidics, Zwijnaarde, Belgium) connected to a nanoAcquity LC system (Waters, Milford, MA, USA). Separation was performed in reverse phase using a linear gradient of mobile phase B (0.1% formic acid in 98% acetonitrile) from 1% to 40% in 80 min, followed by a steep increase to 100% mobile phase B in 5 min. After 5 min at 100% mobile phase B, a steep decrease to 1% mobile phase B was achieved in 5 min and maintained for 35 min at a flow rate of 750 nL per min. The LC system was coupled to a Q-Exactive Plus orbitrap mass spectrometer (Thermo Fisher Scientific, Waltham, MA, USA) programmed to acquire in data-dependent mode. The survey scans were acquired in the orbitrap mass analyzer operating at 70,000 (FWHM) resolving power at the mass range of 350–1850 m/z, with a target of 3E6 ions and 100 ms injection time. Precursors were selected “on the fly” for high energy collision-induced dissociation (HCD) fragmentation with an isolation window of 1.6 amu and a normalized collision energy of 28%. A target of 1.7E3 ions and a maximum injection time of 80 min were used for MS/MS. The method was set to analyze the top 20 most intense ions from the survey scan and dynamic exclusion was enabled for 20 s. Tandem mass spectra were processed using MaxQuant software version 1.6.7.0. Proteins were identified using the Andromeda search engine and using the *O. volvulus* reference database (WormBase, https://www.wormbase.org) (accessed on 3 August 2021). Results were uploaded in Perseus software (version 1.6.7.0), where the data matrix was filtered by removing protein IDs identified by site, potential contaminants, and decoy reverse sequences, where applicable.

### 2.4. In Silico Screening of Signal Peptide, Homology and Antigenicity Predictions

The protein sequences were then retrieved in FASTA format from the WormBase server using their respective accession numbers. The sequences were then subjected to (1) signal peptide predictions using SignalP 5.0 (http://www.cbs.dtu.dk/services/SignalP/) (accessed on 13 September 2021), (2) Homology BLAST using UniProt BLAST (https://www.uniprot.org/blast/) (accessed on 13 September 2021) and the NCBI BLASTp tool (https://blast.ncbi.nlm.nih.gov/Blast.cgi) (accessed on 13 September 2021) (3) antigenicity prediction in the VaxiJen 2.0 server (http://www.ddg-pharmfac.net/vaxijen/VaxiJen/VaxiJen.html) (accessed on 13 September 2021) and ANTIGENpro server (http://scratch.proteomics.ics.uci.edu/) (accessed on 13 September 2021). Only proteins that were predicted to have a signal peptide, low conservation (<30% homology) in related nematodes, not conserved in humans, and antigenic (threshold of 0.5) were selected for further analysis.

### 2.5. Transmembrane Domain and Linear B-Epitope Prediction

All selected proteins were screened for the presence of transmembrane domains in the TMHMM server (http://www.cbs.dtu.dk/services/TMHMM/) (accessed on 19 September 2021) and TOPCONS server (https://topcons.cbr.su.se/servers) (accessed on 19 September 2021). The BCpreds (http://ailab-projects1.ist.psu.edu:8080/bcpred/) (accessed on 21 September 2021) server was then employed for the prediction of linear B-epitopes from the proteins. BCpreds predicts continuous B-cell epitopes using a support vector machine (SVM) and a subsequent kernel (SSK) approach, with a prediction accuracy of 74.57 [33].

### 2.6. Multiepitope Antigen Construction, Antigenicity, Physicochemical Properties and Solubility Prediction

Following prediction of suitable B-epitopes outside the transmembrane domains, screening in ANTIGENpro and Vaxijen 2.0 servers, a maximum of 3 top scoring B-epitopes per protein were selected to construct the multiepitope antigen. These epitopes were linked together with a flexible GSGSG linker to maximize epitope stability and recognition [21,34]. Methionine and 6xHis tag were added to the N and C terminus respectively to obtain the sequence of OvMCBL02 multiepitope antigen. Using Vaxijen 2.0, the antigenicity of OvMCBL02 was predicted and its solubility was predicted by the Protein-Sol server (https://protein-sol.manchester.ac.uk/) (accessed on 21 September 2021). Furthermore, the physico-chemical properties of OvMCBL02 multi-epitope antigen were assessed in the ExPASY ProtParam server (http://web.expasy.org/protparam/) (accessed on 21 September 2021) [35].

### 2.7. Secondary Structure Prediction

RaptorX server (http://raptorx.uchicago.edu/StructurePropertyPred/predict/) (accessed on 22 September 2021) was employed for prediction of the secondary structure of OvMCBL02 multiepitope antigen. RaptorX server is a web server that predicts a protein sequence’s structural property without making use of templates. It surpasses other servers, particularly for proteins with few or no PDB homologs and a sparse sequence profile. This server uses the Deep Convolutional Neural Fields an emerging machine learning model to predict secondary structure, solvent accessibility, and disorder regions all at once [36].

### 2.8. Serological Assessment of OvMCBL02 Multiepitope Antigen

The amino acid sequence of OvMCBL02 multiepitope antigen was sent to GenScript, (Piscataway, NJ, USA) for codon optimization, synthesis, cloning in a pET30a vector, expression, and purification. Total IgG responses to OvMCBL02 multiepitope antigen were explored by indirect ELISA using serum samples from both infected and non-infected individuals. Optimal antigen and antibody concentrations were determined using the checkerboard titration method [37]. Maxisorp 96-well microtiter plates (Nunc, Roskilde, Denmark) coated with 50 µL of 2 µg/mL purified OvMCBL02 multiepitope antigen were incubated at room temperature for 2 h. The plates were washed thrice at 5 min intervals with wash buffer (PBS + 0.5% Tween 20) and blocked overnight at 4 °C using 50 µL of 5% Bovine Serum Albumin (BSA) (Sigma, St. Louis, MI, USA) as blocking solution. Microtiter plates were again washed thrice at 5 min intervals and incubated at room temperature for 90 min with 50 µL of the various serum samples as primary antibodies at a dilution of 1:2000. After that, plates were washed in a similar procedure and incubated for 90 min at room temperature with 50 µL of goat anti-human IgG (Fc Specific) peroxidase conjugate (Sigma, St. Louis, MI, USA) as the secondary antibody at a dilution of 1:5000. Thereafter, a final washing was carried out and 50 µL of the chromogenic substrate 3,3′,5,5′-tetramethylbenzidine (TMB, Sigma, St. Louis, MI, USA) was added and the plates were incubated at room temperature for 5 min. After stopping the reactions with 3M hydrochloric acid, the optical densities were measured at 450 nm. Immune responses against IgG4 antigen were determined using serum pools from 10 onchocerciasis patients and 10 European controls. Indirect ELISA was performed as reported with the exception of incubating the pooled serum samples as primary antibodies at a serial dilution from 1:250 to 1:32,000 and using mouse monoclonal anti-human IgG1, IgG2, IgG3 (Sigma, St. Louis, MI, USA) and IgG4 Fc (HRP) antibody (Abcam, Cambridge, UK) as the secondary antibody.

### 2.9. Data Analysis

Data were analyzed using Microsoft Excel 365. The Shapiro–Wilk test was used to determine the normality of distributions. Scatter plots were generated using GraphPad Prism 9.3.1 (La Jolla, CA, USA) and the data were expressed as median with interquartile range. Kruskal–Wallis test followed by Dunn’s test were used for multiple comparisons among 3 groups. Receiver operating curve analysis was used to evaluate total IgG’s discriminating performance. The area under the receiver operating curve were evaluated using the trapezoid method and an optimal cutoff value was selected based on the Youden’s index to obtain the sensitivities, specificities, and 95% confidence intervals for the selected cutoff value. All values were considered statistically significant at *p*-value < 0.05.

## 3. Results

### 3.1. Protein Selection

All nodules obtained from persons residing in the Kombone Health Area had life worms; thus, samples from these individuals were processed for proteomic analysis. Following mass spectrometry (complete data set under analysis for publication), a total of 1392 proteins were expressed in both the adult and microfilaria stages of *O. volvulus*. Signal peptide prediction using the sequences of these proteins in the SignalP 5.0 server predicted 163 proteins to contain signal peptides. Following a BLAST search of all proteins predicted to possess a signal peptide in the UniProt database and BLASTp tool of NCBI, a total of nine proteins were selected based on low sequence identity in related nematodes such as *Wuchereria bancrofti* and *Brugia malayi* (cut off at <30% identity) (Table S1). Six out of the nine proteins were predicted to be antigenic in the VaxiJen 2.0 and ANTIGENpro servers (Table 1). Both TMHMM and TOPCONS servers predicted OVOC8498 to possess a transmembrane domain. The BCpreds server was used to predict linear B-epitopes of variable residue lengths and a maximum of three antigenic linear B-epitopes were selected per protein. For OVOC8498, the linear B-epitopes were selected outside the transmembrane region since it is difficult to express proteins containing transmembrane domains [38].

### 3.2. OvMCBL02 Multiepitope Antigen Construction

**Figure 1:**
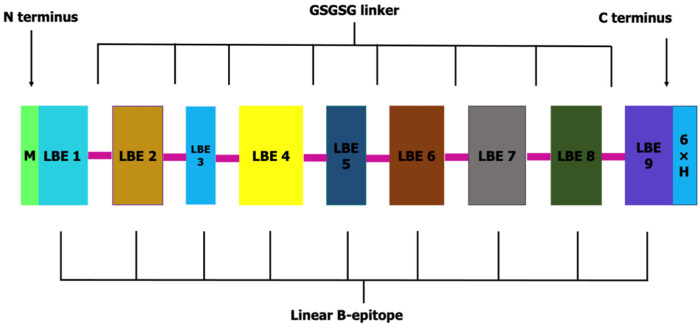
Schematic representation of OvMCBL02 multiepitope antigen with methionine coupled at the N-terminus and 6X His tag to its C-terminus. The various linear B-epitopes (LBE) are represented with different colors.

A total of nine antigenic linear B-epitopes were linked with a GSGSG flexible linker to construct OvMCBL02 multiepitope antigen. Methionine was included at the N-terminus for expression purpose and to ease purification and identification of the multiepitope antigen, a 6xHis tag was attached to the C-terminus (Figure 1).

### 3.3. Antigenicity Prediction, Physicochemical Properties, Solubility and Secondary Structure of OvMCBL02 Multiepitope Antigen

**Figure 2:**
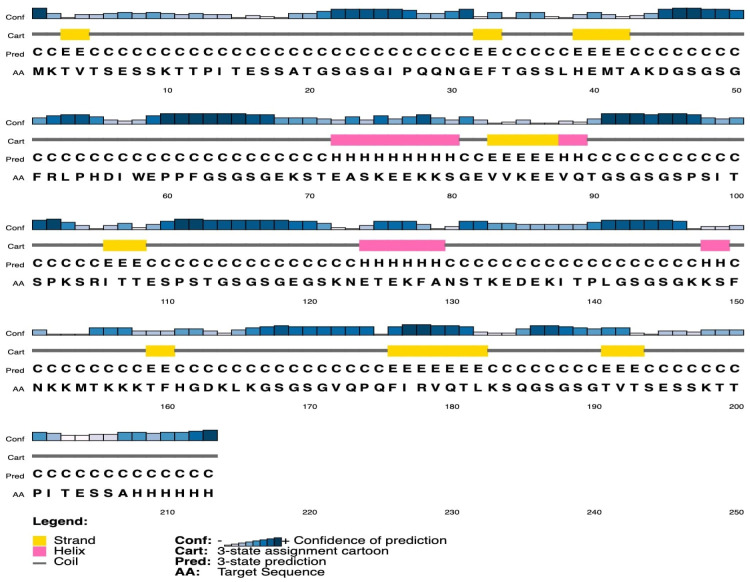
Secondary structure of OvMCBL02 multiepitope antigen predicted using RaptorX server with alpha-helix (8.9%), beta strands (13.1%), and coils (78.0%).

Both ANTIGENpro and Vaxijen 2.0 servers predicted the multiepitope antigen to be antigenic with a score of 0.894453 and 1.0606 respectively. OvMCBL02 multiepitope antigen is composed of 213 amino acids with molecular weight of 22.1 kDa and theoretical pI of 8.96 based on predictions on ExPasy ProtParam. The antigen has an instability index of 39.67 (Cut off = 40.00) which classifies it as stable upon expression. The Grand average of hydropathicity (GRAVY) was predicted to be −1.033, suggesting that the antigen is hydrophilic in nature and can interact with water molecules [39]. Consistently, the protein-Sol server predicted the antigen to be soluble with a score of 0.658. The multiepitope antigen was predicted to contain 8.9% alpha-helix, 13.1% beta-strand, and 78% coil (Figure 2).

### 3.4. Humoral Immune Response to OvMCBL02 Multiepitope Antigen

**Figure 3:**
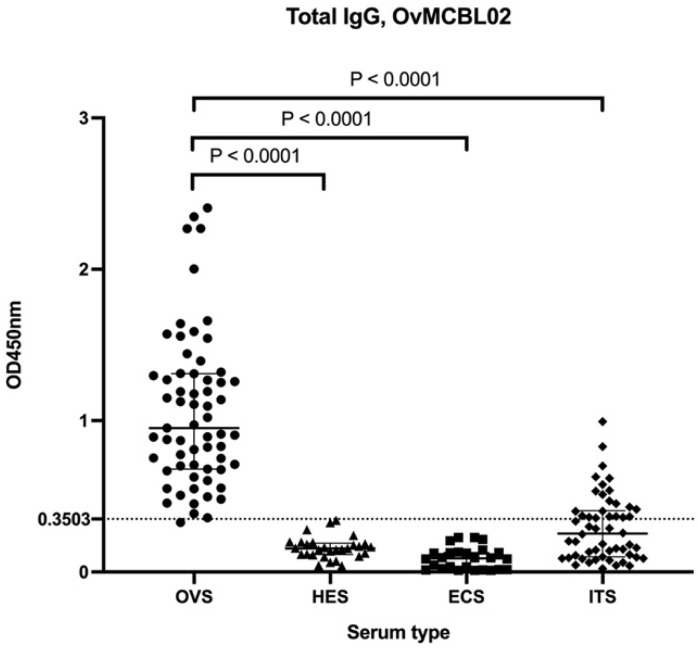
Humoral immune responses to OvMCBL02 multiepitope antigen by ELISA. OVS = *O*. *volvulus* serum (*n* = 63), HES = Hypo-endemic serum (*n* = 29), ECS = European control serum (*n* = 26), ITS = Ivermectin treated serum (*n* = 54). Kruskal Wallis test followed by Dunn’s test was used for multiple comparisons among the groups.

After the multiepitope antigen was synthesized, cloned, expressed, and purified by GenScript, (Piscataway, NJ, USA), its diagnostic potential was evaluated by assessing total IgG responses in indirect ELISA using serum samples from *O. volvulus* infected and non-infected individuals. Total IgG immune responses to OvMCBL02 multiepitope antigen differentiated these two groups of participants. All optical densities were read at 450 nm and the OD values for onchocerciasis serum samples (OVS) compared with those of hypoendemic serum samples (HES) from Rwanda were significantly higher (*p* < 0.0001). Similarly, the OD values for OVS were significantly higher compared with those of European control serum (ECS) (*p* < 0.0001) (Figure 3). The area under the receiver operating curve (AUC) was 0.9995, with a *p* < 0.0001 (Table 2), indicating both high sensitivity and specificity.

Humoral immune responses to OvMCBL02 multiepitope antigen were measured using sera from regions with different levels of onchocerciasis endemicity (*O. volvulus* serum samples from Kombone—OVS and Ivermectin-treated serum samples from Bandjoun—ITS) by ELISA to assess the possibility of using OvMCBL02 as a biomarker to monitor the performance of elimination programmes. The multiepitope antigen could significantly differentiate between OVS and ITS (*p*-value < 0.0001) (Figure 3).

### 3.5. Assessment of Humoral Immune Response of Related Nematode Sera to OvMCBL02 Multiepitope Antigen

**Figure 4:**
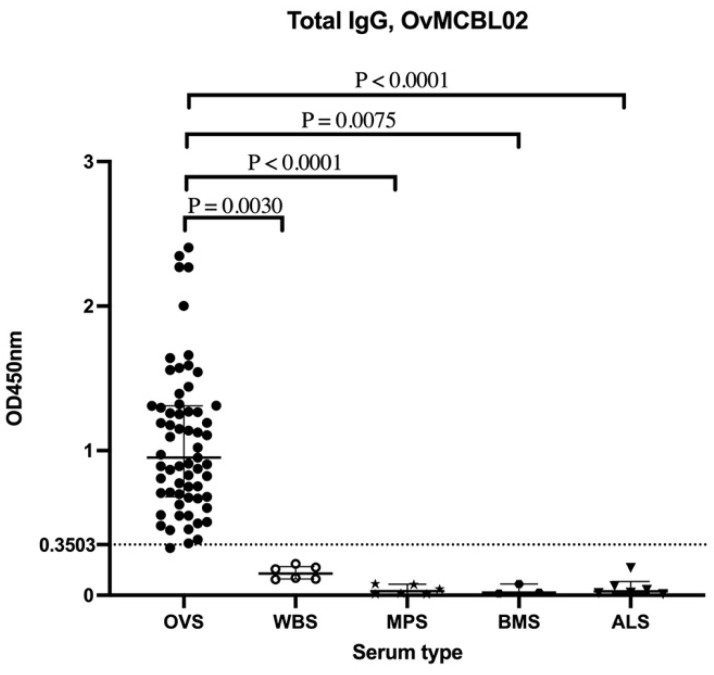
Evaluation of the humoral immune responses of related nematode sera to the OvMCBL02 multiepitope antigen. *O. volvulus* sera (OVS, *n* = 63), *Wuchereria bancrofti* sera (WBS, *n* = 6), *Mansonella perstans* sera (MPS, *n* = 6), *Brugia malayi* sera (BMS, *n* = 3) and *Ascaris lumbricoides* sera (ALS, *n* = 6). Kruskal–Wallis test followed by Dunn’s test were used for multiple comparisons among the groups.

The probability of having OvMCBL02 chimeric antigen to cross react in individuals infected with related nematodes such as *W. bancrofti, M. perstans, B. malayi*, and *A. lumbricoides* was assessed by ELISA. It was observed that OvMCBL02 test could significantly distinguish *O. volvulus* sera from sera of the aforementioned related nematodes (Figure 4).

### 3.6. Total IgG, IgG1, IgG2 and IgG3 but Not IgG4 Subclass Responded Positively to OvMCBL02 Multiepitope Antigen

**Figure 5:**
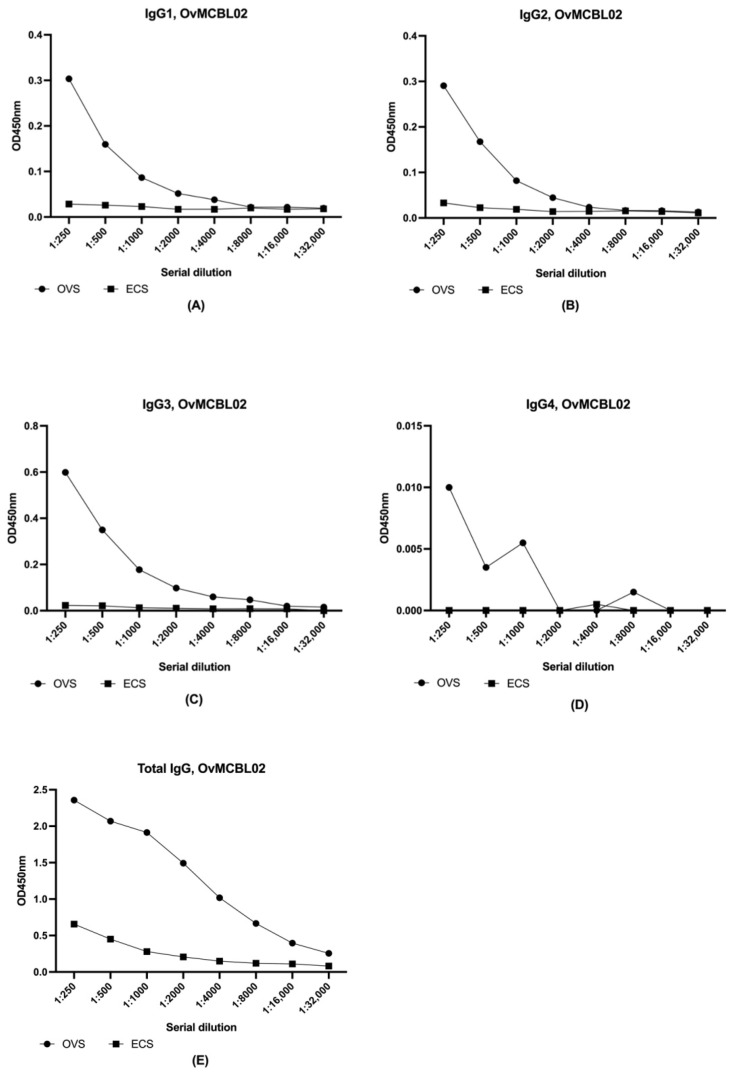
Measurement of IgG subclasses and total IgG response to OvMCBL02 multiepitope antigen using sera pools from infected (OVS, *n* = pool of 10 infected serum samples) and non-infected individuals (ESC, *n* = pool of 10 control serum samples). Microtiter plates were coated with purified antigen for 2 h and blocking was performed overnight. Plates were incubated with serum pools from either OVS or ECS at different dilutions (1:250 to 1:32,000) followed by incubation with (**A**) anti-human IgG1 (Fc-specific) antibody (**B**), mouse monoclonal anti-Human IgG2, (**C**) mouse monoclonal anti-Human IgG3, (**D**) mouse monoclonal anti-Human IgG (HRP), or (**E**) goat anti-human IgG peroxidase conjugate as the secondary antibody. TMB was used for revelation and optical densities read at 450 nm. OD values were plotted against the different serum types.

IgG subclass immune responses to OvMCBL02 multiepitope antigen were investigated using serum pools for both infected (OVS) and uninfected individuals (ECS). Although IgG4 measurements have been reported to accounts for up to 95% of the IgG response in filarial infections [40], OvMCBL02 multiepitope antigen did not react with the IgG4 subclass as shown on the serial dilution curve (Figure 5D) and was unable to differentiate between pools of sera from uninfected and infected individuals. On the other hand, total IgG, IgG1, IgG2, and IgG3 were evaluated in the same serially diluted serum sample pools and OvMCBL02 multiepitope antigen could significantly differentiate *O. volvulus* infected from uninfected serum samples (Figure 5A–C,E).

## 4. Discussion

The diagnosis of onchocerciasis by detecting the parasite in humans has been challenging and variable, with clinical presentations ranging from being evident and helpful in heavily infected patients (e.g., observation of microfilaria in the anterior segment of the eye [41], presence of microfilaria in skin-snip biopsies [42], and identification of palpable nodules harboring adult worms [43]) to being relatively insensitive in persons having lower parasite loads [9]. Currently, the preferred epidemiological approach to diagnose *O. volvulus* infection utilizing sera from humans relies on the detection of an antibody response to the Ov-16 antigen [44,45]. However, this test has moderate sensitivity ranging between 60%–80% [18] and cannot distinguish between current and past infections [46], which renders it limited in the diagnosis of onchocerciasis. Thus, new diagnostic tools are needed to effectively diagnose onchocerciasis in the era of onchocerciasis elimination in Africa. The value of developing tests that incorporate a wider range of antigens have been highlighted previously by our group [46] and in a study from Yemen [47], where the use of additional antigens increased the detection of individuals exposed to *O. volvulus.* Thus, in this study, nine linear B-epitopes from six proteins were fused to form a multiepitope antigen (OvMCBL02).

The multiepitope antigen was predicted to be antigenic, implying that it can react specifically with the functional binding site of its complementary antibody [48]. Furthermore, the OvMCBL02 multiepitope antigen was predicted to be soluble and stable upon expression and this was confirmed during the expression and purification stages of the multiepitope antigen. Prediction of the physicochemical properties of the OvMCBL02 multiepitope antigen revealed its molecular weight to be 22.1 KDa. This low molecular weight of the multiepitope antigen is of importance as it has been reported that the use of low-molecular-weight antigens in diagnosis increases assay specificity [49]. Its theoretical pI was 8.96, implying the multiepitope antigen is alkaline in nature, an important feature beneficial for its purification by ion-exchange chromatography and isoelectric focusing procedures. The predicted secondary structure of OvMCBL02 multiepitope antigen revealed that the protein is made up mostly of coils (78.0%). Natively unfolded protein regions and alpha-helical coiled-coils peptides are useful for the design of epitope-based diagnostic tests as these two forms are capable to fold into their native structure and be recognized by antibodies naturally induced by parasites [50].

To evaluate the diagnostic potentials of OvMCBL02 multiepitope antigen, total IgG responses to the chimeric antigen were validated using *O. volvulus*-infected sera (OVS) and uninfected sera (HES and ECS). Assessment of the geographic distribution of onchocerciasis in Cameroon revealed that onchocerciasis is endemic in all the ten regions of Cameroon with approximately 60% of the population living in high-risk areas for the disease [31]. Furthermore, assessment of communities which have been receiving preventive chemotherapy for close to 20 years revealed the continuous spread of the parasite in some communities [51,52]. The OvMCBL02 test is an antibody-based test which is unable to differentiate between past and current infection; thus, control samples were obtained from Rwanda and Europe rather than Cameroon to exclude the possibility of including healthy individuals that may have been infected with the parasite in the past. Consistently, we were unable during our previous research activities to identify individuals who fully responded negative to antibody tests using other antigens, which is the reason why serum samples from a country hypoendemic for onchocerciasis such as Rwanda was the best control option [46]. The OvMCBL02 multiepitope antigen was able to significantly differentiate between serum from infected and uninfected individuals. After prioritizing the specificity of the OvMCBL02 test, a cut-off value of 0.3503 was chosen corresponding to 100.0% specificity and 98.4% sensitivity. At this cutoff value, the OvMCBL02 test was able to differentiate between infected individuals and patients who had been undergoing treatment. These results are of importance in the assessment of onchocerciasis treatment strategy since this will provide information on the response status of the parasite on ivermectin when administered and it can be used to certify an individual to be free from the disease after receiving ivermectin for more than a decade. At the selected cut-off value of 0.3503, no positive sample was found in any of the control groups. However, 21 positive samples were found in the ivermectin-treated group. These 21 positive samples in the ITS group may have been from individuals who have not yet cleared the parasite despite being on treatment or who were infected in the past. The OvMCBL02 test is an antibody-based test that is unable to distinguish between active and passive infections. In addition, cross reactions resulting from possible coinfections with *Loa loa*, a closely related nematode of *O. volvulus* should not be excluded. However, this will be addressed in our future investigations. OvMCBL02 multiepitope antigen did not cross react with serum samples from related nematodes such as *W. bancrofti*, *B. malayi,* *M. perstans* and *A. lumbricoides* and hence may be employed for the specific detection of onchocerciasis in zones where *O. volvulus* is co-endemic with these related nematodes. Due to the small sample size used in the analysis of cross reactivity, our study is not sufficiently powered to guarantee the superior specificity of OvMCBL02 test; thus, it is necessary to use a higher sample size and also include other related nematodes during further characterization of OvMCBL02 multiepitope antigen.

It has been reported that IgG4 subclass responses are the domineering humoral immune responses against parasite antigens [53]. Using serum pools from both infected and uninfected individuals, IgG4 responses were weak with OvMCBL02 multiepitope antigen and could not significantly differentiate infected pooled sera from uninfected pooled sera. On the other hand, total IgG, IgG1, IgG2, and IgG3 responses to OvMCBL02 could significantly discriminate between these set of serum samples. A possible explanation why there is no IgG4 response to the OvMCBL02 multiepitope antigen might be due to the difference between conformational epitopes on intact surface antigens. This hypothesis is in line with reports from other studies on Ov-20 immunodominant antigen, where only the intact protein could be recognized by IgG4 antibodies, while IgG1, IgE, and IgM antibodies were shown to also bind smaller fragments of the antigen [54]. Considering that IgG3 is more reactive, the OvMCBL02 multiepitope antigen could also be investigated for its role as a vaccine candidate against onchocerciasis since IgG3 immune responses have been reported to be defensive against the parasite [55].

In conclusion, the OvMCBL02 multiepitope antigen was demonstrated to be immunogenic with a potential of serving as a diagnostic antigen (high sensitivity and specificity) for onchocerciasis. Thus, further characterization of the OvMCBL02 multiepitope antigen and comparison of the OvMCBL02 test with the Ov-16 test recommended by the WHO may render it an additional member of the diagnostic toolkit for the elimination of onchocerciasis.

## Figures and Tables

**Table 1:** Antigenicity of low conserved proteins in related nematodes and selected linear B-epitopes.

S/N	Protein ID	Antigenicity (Cut off > 0.4999)			Linear B-Epitopes Selected	
		ANTIGENpro	Vaxijen 2.0	Remarks	Linear B-Epitopes	Antigenicity on Vaxijen 2.0
1	OVOC7606	0.493181	0.3129	Non antigenic	None	-
2	OVOC9989	0.247895	0.4514	Non antigenic	None	-
3	OVOC10207	0.377321	0.2670	Non antigenic	None	-
4	OVOC5574	0.936181	0.4886	May be antigenic	IPQQNGEFTGSSLHEMTAKD (LBE 2)	0.7725
5	OVOC5909	0.605038	0.7636	Antigenic	TVTSESSKTTPITESSA (LBE 9)	1.0945
					KTVTSESSKTTPITESSAT (LBE 1)	1.0717
					SPSITSPKSRITTESPST (LBE 5)	1.1604
6	OVOC8498	0.678848	0.8364	Antigenic	FRLPHDIWEPPF (LBE 3)	0.8433
7	OVOC8529	0.844335	0.5419	Antigenic	KKSFNKKMTKKKTFHGDKLK (LBE 7)	0.7077
8	OVOC8936	0.732818	0.5409	Antigenic	VQPQFIRVQTLKSQ (LBE 8)	0.6061
9	OVOC10037	0.844335	1.1807	Antigenic	EGSKNETEKFANSTKEDEKITPL (LBE 6)	1.1520
					EKSTEASKEEKKSGEVVKEEVQT (LBE 4)	1.4237

LBE = Linear B-epitope position on the designed multiepitope antigen construct.

**Table 2:** Receiver operating curve (ROC) values for IgG responses to OvMCBL02 multiepitope antigen and diagnostic accuracy parameter.

Total IgG		
**ROC Curve Analysis**	ROC curve area (AUC)	0.9995
	95% CI of AUC	0.9976 to 1.000
	*p*-value (against AUC = 0.5)	<0.0001
**Diagnostic Accuracy Parameter**	Cut off value	0.3503
	Sensitivity (%) (95% CI)	98.4 (91.54% to 99.92%)
	Specificity (%) (95% CI)	100.0 (88.30% to 100.00%)

## Data Availability

The datasets generated during this study are available from the corresponding authors on reasonable request.

## Supplementary Materials

The following supporting information can be downloaded at: https://www.mdpi.com/article/10.3390/diagnostics12061440/s1, Table S1: Homology prediction of proteins with signal peptides.
