# Supplementary material for: Immunoinformatics Design and Assessment of a Multiepitope Antigen (OvMCBL02) for Onchocerciasis Diagnosis and Monitoring
Source: Diagnostics (Basel). 2022 Jun 11;12(6):1440. doi: 10.3390/diagnostics12061440 (PMC9221995; doi:10.3390/diagnostics12061440)
Supplement: Supplementary file 1 [file diagnostics-12-01440-s001.zip › diagnostics-1707905-supplementary.pdf]

diagnostics

Article

Immunoinformatics Design and Assessment of a Multiepitope Antigen (OvMCBL02) for Onchocerciasis Diagnosis and Monitoring

Table S1: Homology prediction of proteins with signal peptides

Homology blast	Protein ID
Not conserved in related nematodes (<30% similarity in both UniProt and BLASTp databases)	OVOC7606, OVOC10207, OVOC5909, OVOC8529, OVOC8936, OVOC9989, OVOC10037, OVOC5574, OVOC8498
Conserved in related nematodes (>30% similarity in both UniProt and BLASTp databases)	OVOC9892, OVOC9855, OVOC9725, OVOC9497, OVOC9403, OVOC9322, OVOC9058, OVOC8895, OVOC8585, OVOC8491, OVOC8413, OVOC8359, OVOC82, OVOC8118, OVOC8072, OVOC7872, OVOC7843, OVOC7777, OVOC7748, OVOC7660, OVOC7454, OVOC7453, OVOC7373, OVOC7328, OVOC727, OVOC6997, OVOC6944, OVOC693, OVOC68, OVOC6669, OVOC6639, OVOC6422, OVOC6203, OVOC10067, OVOC10263, OVOC10476, OVOC10654, OVOC10802, OVOC10602, OVOC10872, OVOC10939, OVOC11417, OVOC11487, OVOC11516, OVOC11845, OVOC11951, OVOC12274, OVOC12359, OVOC12449, OVOC1287, OVOC1536, OVOC1709, OVOC193, OVOC2249, OVOC2366, OVOC243, OVOC2493, OVOC2683, OVOC281, OVOC2991, OVOC3048, OVOC3290, OVOC3523, OVOC3864, OVOC387, OVOC4153, OVOC4177, OVOC4230, OVOC4680, OVOC4952, OVOC 5288, OVOC5386, OVOC5967, OVOC6112, OVOC6381, OVOC6908, OVOC6942, OVOC7011, OVOC7138, OVOC7265, OVOC7292, OVOC7617, OVOC7804, OVOC8052, OVOC8395, OVOC8665, OVOC8677, OVOC89, OVOC8919, OVOC9161, OVOC9325, OVOC9326, OVOC9327, OVOC9592, OVOC9593, OVOC9726, OVOC9818, OVOC9984, OVOC9988, OVOC9990, OVOC10071, OVOC10371, OVOC10382, OVOC1058, OVOC10635, OVOC10769, OVOC10860, OVOC10873, OVOC10910, OVOC11026, OVOC11170, OVOC11411, OVOC6875, OVOC11707, OVOC12067, OVOC12077, OVOC12148, OVOC12472, OVOC12476, OVOC12488, OVOC12544, OVOC12649, OVOC13508, OVOC140, OVOC1512, OVOC12236, OVOC11881, OVOC1662, OVOC1743, OVOC1952, OVOC1978, OVOC2208, OVOC2262, OVOV235, OVOC2424, OVOC2544, OVOC2978, OVOC311, OVOC3120, OVOC3665, OVOC37, OVOC4051, OVOC4100, OVOC4148, OVOC4447, OVOC4636, OVOC4906, OVOC589, OVOC5121, OVOC5169, OVOC5280, OVOC5640, OVOC5935, OVOC6038
